# Decent work perception and burnout in pediatric nurses: the mediating effect of basic psychological needs based on self-determination theory

**DOI:** 10.3389/fpubh.2026.1901864

**Published:** 2026-07-24

**Authors:** Hao Xu, Min Xu

**Affiliations:** 1 Department of Pediatric Critical Care Medicine Nursing, West China Second University Hospital, Sichuan University, Chengdu, Sichuan, China; 2 Key Laboratory of Birth Defects and Related Diseases of Women and Children (Sichuan University), Ministry of Education, Chengdu, Sichuan, China

**Keywords:** basic psychological need, burnout, decent work perception, occupational health, pediatric nurse

## Abstract

**Background:**

Grounded in self-determination theory, basic psychological needs are intrinsic factors that regulate employees' occupational mental health. Pediatric nurses face heavy work pressure and high levels of emotional labor, leading to prevalent occupational burnout. This study aimed to explore the association between decent work perception and burnout among pediatric nurses and examine the mediating role of basic psychological needs.

**Methods:**

This multicenter cross-sectional survey recruited 355 pediatric nurses from five tertiary hospitals across Sichuan Province. Validated self-report instruments including the Decent Work Perception Scale, Basic Psychological Need Scale and Maslach Burnout Inventory-General Survey were used to collect research data. Pearson product-moment correlation coefficients were used to test the correlations among core variables. On this basis, structural equation modeling was adopted to verify the hypothesized mediating effect paths.

**Results:**

Pearson correlation analysis showed that pediatric nurses' decent work perception was positively correlated with basic psychological needs (*r* = 0.69, *p* < 0.001). In contrast, decent work perception was negatively associated with occupational burnout (*r* = −0.56, *p* < 0.001), and basic psychological needs also demonstrated a significant negative correlation with burnout (*r* = −0.65, *p* < 0.001). Mediation analysis confirmed that basic psychological needs partially mediated the relationship between decent work perception and occupational burnout. The mediating effect value was −0.36 (95% *CI*: −0.47 to −0.24), accounting for 70.01% of the total effect of decent work perception on burnout.

**Conclusion:**

Perceived decent work was negatively associated with occupational burnout among pediatric nurses, and this relationship was partially mediated by basic psychological need satisfaction. Improving workplace decency and meeting nurses' psychological demands may help relieve burnout and stabilize the pediatric nursing workforce.

## 1 Introduction

With the continuous improvement of modern public health service systems, the occupational mental health of healthcare workers has gradually become a major social concern across global health fields. Clinical nurses face long-term heavy workloads and chronic work pressure, making them particularly vulnerable to negative emotional weariness (1). Widespread occupational burnout among healthcare workers not only impairs individual physical and mental wellbeing but also poses potential risks to the stable operation of medical services and the sustainable development of public health systems (2). Among various clinical departments, pediatric nursing represents a core yet high-pressure branch of clinical nursing work, involving unique occupational stressors distinct from those in adult care settings (3). Pediatric patients often lack full verbal expression skills and show poor cooperation during diagnosis and treatment. Meanwhile, their parents tend to have excessively high emotional expectations and medical anxiety, which further exacerbates the interpersonal stress experienced by frontline pediatric nurses (4, 5). Additionally, pediatric departments are characterized by intensive shift work, frequent emergency treatments, insufficient staffing, and limited career advancement channels in most tertiary and primary hospitals (6, 7). Under the long-term accumulation of multiple occupational stressors, pediatric nurses have become a high-risk group for occupational burnout within the global nursing workforce (8).

### 1.1 Decent work perception and burnout

Occupational burnout is a persistent psychophysiological stress syndrome induced by chronic workplace stress that remains unrelieved over time, mainly manifesting as emotional exhaustion, depersonalization, and reduced personal accomplishment (9). A substantial body of longitudinal nursing research has verified that severe occupational burnout not only damages nurses‘ sleep quality, mental health, and physical immune function but also impairs their clinical nursing decision-making ability and reduces patient satisfaction with nursing care (10, 11). Furthermore, burnout serves as a critical predictor of nurses' turnover intention, job avoidance behavior, and loss of clinical nursing talent, thereby directly threatening the stability of pediatric nursing teams and the safety of pediatric medical care, while also restricting the high-quality and sustainable development of pediatric nursing services (12–14). Therefore, exploring modifiable protective factors, both psychological and occupational environmental, to alleviate occupational burnout among pediatric nurses holds important clinical practical value and public health significance.

Decent work perception, an emerging subjective occupational psychological evaluation indicator in the field of occupational health psychology in recent years, refers to nurses' overall cognitive and emotional appraisal of multiple job dimensions, including reasonable remuneration, safe working environment, guaranteed occupational rights, harmonious interpersonal relationships, and fair career development opportunities (15). The International Labor Organization (ILO) has long emphasized that decent work constitutes the core guarantee for employees' occupational wellbeing, and improving employees' perception of decent work is a key organizational intervention to reduce negative workplace emotions (16). Existing relevant studies have confirmed that decent work perception negatively predicts various negative occupational outcomes among healthcare workers: those with higher levels of decent work perception report lower occupational stress, less workplace anxiety, and stronger job engagement (17, 18). Nevertheless, most current studies have focused on general clinical nurses or emergency nurses, leaving targeted research on pediatric nurses, who work in special clinical scenarios, still insufficient. Moreover, the internal psychological relational patterns underlying the association between perceived decent work and burnout in pediatric nurses remain underexplored, which constrains efforts to refine hospital human resource management strategies through improvements to workplace conditions conducive to decent work.

### 1.2 The mediating effect of basic psychological needs based on self-determination theory

Self-Determination Theory (SDT) provides a mature theoretical framework for explaining the mediating pathway linking the external occupational environment, individual psychological cognition, and occupational behavioral outcomes, and this theory is widely applied in occupational health and nursing psychology research (19, 20). A large number of cross-sectional observational studies grounded in SDT have reported consistent correlational evidence that basic psychological needs sit between workplace contextual factors and employees' work-related emotional states, which lays a theoretical foundation for exploring how these three variables are mutually associated in nursing cohorts. The job demands-resources (JD-R) model primarily emphasizes the direct buffering effects of job demands and job resources, whereas SDT elucidates the internalization mechanisms underlying intrinsic motivation. The present study adopts SDT as its theoretical framework to investigate how external decent work conditions are transformed into internal psychological experiences to alleviate occupational burnout. By doing so, it supplements empirical evidence from the perspective of micro-level motivational processes, thereby extending the predominantly macro-level occupational resource perspective inherent in the JD-R model. The basic psychological needs sub-theory, one of the key branches of SDT, posits that all individuals possess three innate and universal basic psychological needs: autonomy, competence, and relatedness (21). Favorable external environmental stimuli do not directly affect individuals' occupational attitudes and behaviors; rather, they exert indirect effects through the satisfaction of internal basic psychological needs (22, 23). Specifically, a high-quality decent work environment can grant nurses autonomous working autonomy, recognize their professional competence, and foster harmonious colleague–patient interpersonal relationships, thereby fully meeting their three basic psychological needs. When basic psychological needs are adequately satisfied, individuals generate stable internal work motivation, buffer the negative impact of chronic workplace stress, and further reduce the occurrence of occupational burnout (24). Conversely, elaborate that chronic need frustration is a primary psychological correlate triggering emotional exhaustion, depersonalization, and reduced personal accomplishment under SDT. Suboptimal perceptions of decent work may give rise to sustained dissatisfaction of fundamental psychological needs, thereby plunging nurses into a state of emotional depletion and ultimately exacerbating the manifestation of burnout symptoms (25). In line with existing empirical evidence, previous studies have verified that basic psychological needs play a significant mediating role between occupational environmental factors and negative occupational emotions among nurses (23, 26). Such observed correlational features imply that basic psychological needs may serve as an intermediate associational variable connecting workplace perceptions and adverse occupational emotional outcomes, which motivates the current research to examine the interconnected relationships among decent work perception, basic psychological needs and burnout within a single analytical framework. However, few studies have incorporated decent work perception, basic psychological needs and occupational burnout within a unified theoretical framework to explore the intercorrelations of these three variables among pediatric nurses. Moreover, the existing literature tends to treat decent work as an isolated external organizational resource rather than positioning it alongside the three core psychological needs proposed by SDT as components of a unified explanatory chain. Most prior studies have focused on single mediators, without establishing the SDT-grounded pathway tested in the current work.

### 1.3 The present study

Based on SDT and prior empirical studies, this study develops a mediation model with basic psychological needs as the mediating variable. The purpose is to examine the direct impact of decent work perception on occupational burnout among pediatric nurses and to test the mediating role of basic psychological needs in this relationship. The following research hypotheses are proposed: H1: Decent work perception among pediatric nurses has a significant negative correlation with occupational burnout; H2: Decent work perception among pediatric nurses shows a significant positive association with the satisfaction of basic psychological needs; H3: Basic psychological needs are significantly negatively related to occupational burnout; H4: Basic psychological needs mediate the relationship between decent work perception and occupational burnout in pediatric nurses. This cross-sectional investigation addresses an understudied area in existing scholarship related to correlational interplay between perceived decent work, intrinsic psychological experiences, and burnout within pediatric nursing cohorts. Practically, these cross-sectional correlational findings may deliver preliminary contextual references for nurse managers to optimize workplace arrangements and accommodate frontline nurses' fundamental psychological demands. Such organizational adjustments could potentially correspond with diminished self-perceived burnout, weaker voluntary turnover intentions, more consistent staffing in pediatric wards, and maintained high-standard pediatric healthcare delivery.

## 2 Methods

### 2.1 Participants

This cross-sectional observational study was reported in accordance with the STROBE Statement checklist for observational cross-sectional studies. This study was designed as a multicenter observational investigation and conducted via online questionnaire surveys. In line with the latest regional development layout of Sichuan Province, the province is divided into five major economic zones. One tertiary public hospital was purposively selected from each zone, yielding a total of five participating medical institutions, among which two were general hospitals with pediatric wards and the other three were specialized children's hospitals. Convenience sampling was adopted to recruit pediatric nurses working in these hospitals as research participants. The study was conducted from January to March 2026.After obtaining informed consent, the survey QR code or link was shared via nursing workgroups. All items were required to be completed, and duplicate responses were prohibited. Inclusion criteria: (a) registered pediatric nurses with a valid license; (b) at least one year of pediatric nursing experience; (c) voluntary participation. Exclusion criteria: (a) nurses on external training, standardization training, or internships; (b) those on maternity or sick leave; (c) non-clinical nursing positions. A total of 380 questionnaires were distributed. After excluding those with pediatric clinical nursing experience of less than one year (*n* = 8), non-clinical nursing assignments (*n* = 7), standardized residency training status (*n* = 5), extended sick or maternity leave (*n* = 4), and excessively short completion time (*n* = 1), 355 valid responses were retained, yielding a valid response rate of 93.42%. A sensitivity power analysis conducted with G^*^Power (27) showed that this sample size was sufficient to detect an effect size of *f* ^2^ = 0.021 (α = 0.05, power = 0.80).

### 2.2 Measures

#### 2.2.1 Decent work perception scale

Decent work perception was assessed using a scale developed by Mao et al. (28) specifically for nurses. This 16-item instrument covers five dimensions: work reward, job position, career development, professional recognition, and work climate. Each item is rated on a five-point Likert scale from 1 (strongly disagree) to 5 (strongly agree), with higher total scores indicating a stronger perception of decent work. In this study, the scale showed acceptable internal consistency, with a Cronbach's α of 0.86. Confirmatory factor analysis supported good model fit: χ^2^/df = 2.03, RMSEA = 0.054 [90% *CI* (0.043, 0.065)], CFI = 0.95, TLI = 0.94, and SRMR = 0.059. Standardized factor loadings for all items ranged from 0.48 to 0.91, all exceeding the commonly accepted threshold of 0.40.

#### 2.2.2 Basic psychological need scale

The scale developed by Guardia et al. (29) contains nine items assessing autonomy, competence, and relatedness. Each item is rated on a seven-point Likert scale from 1 (strongly disagree) to 7 (strongly agree), with higher scores indicating higher levels of need satisfaction. In this study, the Cronbach's α for the scale was 0.81. The three-factor model demonstrated acceptable fit: χ^2^/df = 4.14, RMSEA = 0.094 [90% *CI* (0.075, 0.114)], CFI = 0.94, TLI = 0.92, and SRMR = 0.059. Standardized factor loadings for all items ranged from 0.45 to 0.89, all exceeding the commonly accepted threshold of 0.40.

#### 2.2.3 Burnout scale

Burnout was assessed using the Maslach Burnout Inventory – General Survey (30), a widely used instrument in this field. The scale consists of 16 items across three subdomains: emotional exhaustion, cynicism, and reduced personal accomplishment. Items are rated on a seven-point scale from 0 (never) to 6 (every day), with the personal accomplishment subdomain reverse-scored. Higher overall scores indicate greater burnout severity. In this study, the scale showed good internal consistency, with a Cronbach's α of 0.91. Confirmatory factor analysis indicated acceptable model fit: χ^2^/df = 4.45, RMSEA = 0.098 [90% *CI* (0.088, 0.109)], CFI = 0.93, TLI = 0.92, and SRMR = 0.060. Standardized factor loadings for all items ranged from 0.63 to 0.92, all exceeding the commonly accepted threshold of 0.40.

### 2.3 Statistical analysis

Statistical analyses were performed using jamovi 2.3 (The jamovi project, Sydney, Australia) for reliability and validity, SPSS 26.0 (IBM Corp., Armonk, NY, USA) for descriptive statistics and correlations, and Mplus 8.3 (Muthén & Muthén, Los Angeles, CA, USA) for structural equation modeling (SEM). To evaluate the common method bias in self-reported data, both Harman's single-factor test and a confirmatory factor analysis (CFA) model comparison approach (comparing a one-factor model against the proposed measurement model) were employed. Prior to the main analyses, preliminary assumption testing was conducted, including assessments of linearity, residual normality, homoscedasticity, and multicollinearity. Multicollinearity was examined using the variance inflation factor (VIF), with a threshold of VIF < 10 indicating no substantial collinearity among predictors. For the SEM analysis, missing data were handled using full information maximum likelihood (FIML) estimation. Given the continuous nature of the observed indicators and the assumed multivariate normality of the data, the model was estimated using maximum likelihood (ML) estimation. Model fit was evaluated using a combination of absolute and incremental fit indices: the chi-square test (χ^2^), the comparative fit index (CFI), the Tucker–Lewis index (TLI), the root mean square error of approximation (RMSEA) with its 90% confidence interval, and the standardized root mean square residual (SRMR). Acceptable model fit was defined as CFI and TLI ≥ 0.90, RMSEA ≤ 0.08, and SRMR ≤ 0.08. To test the mediation hypotheses, the significance of indirect effects was assessed using bias-corrected bootstrapping with 5,000 resamples, and 95% confidence intervals were computed for all indirect, direct, and total effects. All significance tests were two-tailed with an alpha level of 0.05.

## 3 Results

### 3.1 Demographic characteristics

The sample comprised 320 females (90.14%) and 35 males (9.86%). Participants' ages ranged from 21 to 58 years (*M* = 31.04, *SD* = 6.65). In terms of educational background, six participants (1.69%) had completed secondary technical school, 80 (22.54%) held an associate degree, 264 (74.37%) held a bachelor's degree, and five (1.41%) had earned a master's degree or higher. Years of work experience ranged from 1 to 37 years (*M* = 8.97, *SD* = 7.21). Details are presented in Table 1.

**Table 1:** Demographic and occupational characteristics of the sample (*N* = 355).

Variable	Classification	Number	Percentage (%)
Gender	Male	35	9.86
	Female	320	90.14
Age	≤ 25	68	19.15
	26–35	218	61.41
	36–45	51	14.37
	≥ 46	18	5.07
Education background	Technical secondary school	6	1.69
	Junior college	80	22.54
	Bachelor's degree	264	74.37
	Master's degree or above	5	1.41
Professional title	Nurse	70	19.72
	Senior nurse	187	52.68
	Supervisor nurse	86	24.23
	Associate chief nurse or above	12	3.38
Working years in pediatric nursing	1–5	156	43.94
	6–10	96	27.04
	11–20	89	25.07
	≥21	24	6.76
Marital status	Unmarried	130	36.62
	Married	218	61.41
	Widowed	7	1.97
Monthly income (CNY)	≤ 3,000	26	7.32
	3,000–5,000	91	25.63
	5,000–10,000	192	54.08
	≥10,000	46	12.96
Night shift schedule	Yes	284	80.00
	No	71	20.00
Employment type	Permanent establishment	54	15.21
	Personnel agency contract	7	1.97
	Labor contract	294	82.82
Administrative position	Yes	32	9.01
	No	323	90.99

### 3.2 Common method bias

To assess potential common method bias in the questionnaire data, both Harman's single-factor test and a factor model comparison approach were employed (31). First, the Harman's test revealed that an unrotated exploratory factor analysis yielded eight factors with eigenvalues greater than one, and the largest factor accounted for 31.78% of the total variance—below the recommended threshold of 40%. Second, a comparison was made between a single-factor model (representing a common method factor) and the proposed three-factor model (decent work perception, basic psychological needs, and job burnout). The three-factor model demonstrated a significantly better fit than the single-factor model (Δχ^2^/Δdf = −311.44, *p* < 0.001; ΔCFI = 0.10; ΔTLI = 0.11; ΔRMSEA = −0.014). Taken together, these analytical results indicate that common method bias is unlikely to substantially contaminate the present dataset.

### 3.3 Descriptive statistics and correlations

#### 3.3.1 Demographic differences

No significant gender differences were found in decent work perception (*t* = −0.42, *p* = 0.672) or burnout (*t* = 0.41, *p* = 0.680). Age significantly predicted decent work perception (β = 0.17, *t* = 3.29, *p* < 0.01) and burnout (β = −0.25, *t* = −4.81, *p* < 0.001). Education level showed no significant effects. Work years predicted decent work perception (β = 0.15, *t* = 2.91, *p* < 0.01) and burnout (β = −0.24, *t* = −4.67, *p* < 0.001). Professional title had significant effects on both decent work perception (*F* = 6.08, *p* < 0.001) and burnout (*F* = 3.60, *p* = 0.014). The collinearity diagnostics revealed that the VIF values for all variables fell below five, indicating the absence of severe multicollinearity that could distort the regression estimates. In the present study, preliminary analyses demonstrated that age, length of service, and professional rank were significantly correlated with decent work perception as well as with burnout. These observations are in line with prior studies on nurses' occupational wellbeing, which have repeatedly identified the same variables as robust confounders. On the basis of these empirical and literature-based justifications, we included age, work years, and professional title as covariates in all subsequent regression and mediation models.

#### 3.3.2 Descriptive statistics and partial correlations

Table 2 presents the descriptive statistics for the three main variables and their partial correlations. Pediatric nurses reported moderate levels of decent work perception (*M* = 3.00, *SD* = 0.53), moderately high basic psychological needs (*M* = 4.29, *SD* = 0.67), and moderately high burnout (*M* = 3.02, *SD* = 1.26). Partial correlations (controlling for demographic variables) showed that decent work perception was positively correlated with basic psychological needs (*r* = 0.69, *p* < 0.001) and negatively correlated with burnout (*r* = −0.56, *p* < 0.001). Basic psychological needs were negatively correlated with burnout (*r* = −0.65, *p* < 0.001).

**Table 2:** Descriptive statistics and partial correlations.

Variable	1	2	3	4	5	6	7	8
1 Gender	–							
2 Age	0.13^**^	–						
3 Education	−0.11^*^	0.22^***^	–					
4 Work years	0.15^**^	0.91^***^	0.14^**^	–				
5 Professional title	−0.06	0.77^***^	0.39^***^	0.75^***^	–			
6 Decent work perception	0.02	0.17^**^	0.04	0.15^**^	0.13^*^	–		
7 Basic psychological needs	0.01	0.24^***^	0.05	0.20^***^	0.13^*^	0.69^***^	–	
8 Burnout	−0.02	−0.25^***^	−0.03	−0.24^***^	−0.15^**^	−0.56^***^	−0.65^***^	–

^*^ *p* < 0.05, ^**^p < 0.01, ^***^p < 0.001. Gender: 0 = female, 1 = male; education: 1 = technical secondary school, 2 = associate degree, 3 = bachelor's degree, 4 = master's degree or above; Professional title: 1 = nurse; 2 = senior nurse; 3 = supervisor nurse; 4 = associate chief nurse and above.

### 3.4 Mediation analysis

**Figure 1:**
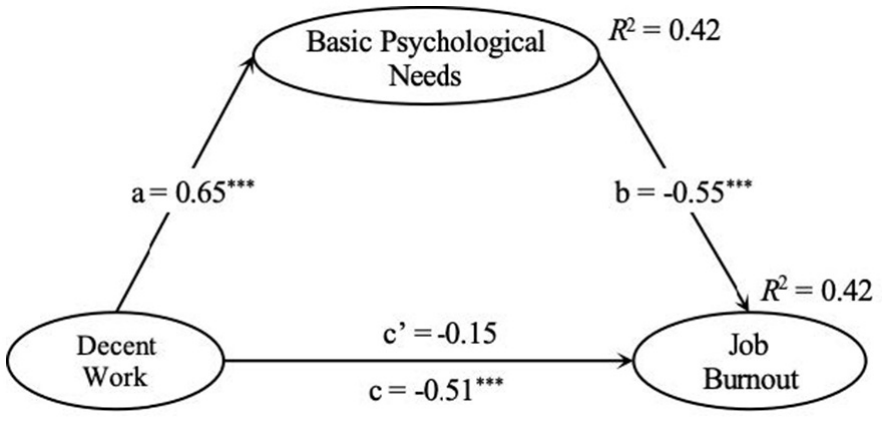
The mediating effect of basic psychological needs between decent work perception and job burnout in pediatric nurses. ^***^p < 0.001. Age, working years and professional title were controlled as three confounding variables in this mediation analysis.

This study employed structural equation modeling (SEM) in Mplus 8.3 to test the mediating effect of basic psychological needs on the association between decent work perception and job burnout in a sample of pediatric nurses. Before incorporating the mediator, the measurement model demonstrated acceptable fit: χ^2^/df = 4.13, CFI = 0.95, TLI = 0.91, RMSEA = 0.094 [90% *CI* (0.072, 0.117)], and SRMR = 0.042. In this initial model, decent work perception exerted a significant negative predictive effect on job burnout [β = −0.58, SE = 0.04, *t* = −14.07, 95% *CI* (−0.66, −0.49), *p* < 0.001]. After incorporating the mediator, the structural equation model continued to demonstrate acceptable fit to the data, χ^2^/df = 4.15, CFI = 0.93, TLI = 0.88, RMSEA = 0.094 [90% *CI* (0.083, 0.106)], and SRMR = 0.063. After controlling for age, working years and professional title, decent work perception exerted a significant positive predictive effect on basic psychological needs [β = 0.65, SE = 0.04, *t* = 19.48, 95% *CI* (0.58, 0.72), *p* < 0.001]. Basic psychological needs had a significant negative predictive effect on job burnout [β = −0.55, *SE* = 0.08, *t* = −6.62, 95% *CI* (−0.71, −0.38), *p* < 0.001]. The direct effect of decent work perception on job burnout was not statistically significant [β = −0.15, *SE* = 0.09, *t* = −1.60, 95% *CI* (−0.33, 0.03), *p* > 0.050; Figure 1]. The mediating effect value was −0.36, with a 95% confidence interval ranging from −0.47 to −0.24. The mediating effect accounted for 70.01% of the total effect (Table 3).

**Table 3:** Mediation analysis of basic psychological needs between decent work perception and job burnout.

Effect	β	*SE*	LLCI	ULCI	*P*	Relative effect
Total effect	−0.51	0.10	−0.70	−0.30	< 0.001	–
Direct effect	−0.15	0.09	−0.33	0.03	0.110	29.99%
Indirect effect	−0.36	0.03	−0.47	−0.24	< 0.001	70.01%

All coefficients are standardized.

## 4 Discussion

Guided by self-determination theory, this cross-sectional study examined the association between decent work perception and burnout among pediatric nurses, with a particular focus on the interrelated correlational roles of basic psychological needs, namely autonomy, competence, and relatedness, in this relationship. After statistically controlling for potential confounding variables including age, clinical tenure, and professional rank, the empirical data offered tentative support for all four proposed hypotheses. Collectively, these cross-sectional findings may provide preliminary empirical evidence that could lend some reference for the design and implementation of targeted human resource management practices, as well as evidence-based mental health promotion programs tailored specifically for pediatric nursing teams.

### 4.1 Demographic differences in research variables

At the descriptive level, pediatric nurses in this sample reported moderate decent work perception and relatively high satisfaction with their basic psychological needs. Meanwhile, their self-reported burnout levels fell within the mild-to-moderate range, indicating a subclinical yet non-negligible burden of occupational stress. Gender and educational attainment had no significant links with the main study variables, yet age, clinical tenure and professional rank correlated significantly with decent work perception and burnout, consistent with prior nursing occupational health evidence (32, 33). Similar workloads, communication pressure and shift arrangements for pediatric nurses may eliminate gender-based disparities in work attitudes and burnout (34, 35). Most participants held bachelor's degrees; limited educational variation and repetitive clinical work may weaken its influence on subjective work evaluations (36). Older and more experienced nurses reported stronger decent work perceptions and milder burnout, supporting our regression results. According to occupational adaptation theory, long-term pediatric clinical practice helps nurses develop coping skills, recognize work value and form positive occupational evaluations (36, 37). Higher professional titles bring greater job autonomy, institutional recognition and promotion opportunities, which can enhance decent work experience and ease burnout (38, 39).

### 4.2 Negative relationship between decent work perception and burnout

Hypothesis 1 was corroborated by the empirical data, which demonstrated a significant inverse relationship between decent work perception and occupational burnout in the pediatric nursing cohort. This pattern of findings echoes those of earlier investigations into nurse wellbeing (17, 40), yet the majority of such studies have been situated within general hospital environments, with limited attention paid to specialized pediatric units. The current research addresses this gap by confirming the protective effect of decent work in a pediatric sample, a population exposed to occupational stressors of notable intensity, including sustained interactions with highly anxious caregivers and frequent management of pediatric emergencies. Within the framework of self-determination theory, decent work perception may be understood as an exogenous motivational resource that establishes the external preconditions for work engagement, thereby exerting an initial buffering effect against burnout that operates in parallel with, and potentially precedes, the internal satisfaction of autonomy, competence, and relatedness.

From a practical standpoint, decent work perception reflects nurses' subjective appraisals of various job-related domains, including remuneration, workplace safety, protection of occupational rights, interpersonal climate, and career advancement opportunities (41). For frontline pediatric nurses, who frequently contend with chronic high workloads, staffing shortages, and constrained promotional avenues, a favorable perception of decent work signifies that the hospital offers competitive compensation and benefits, structured shift schedules, and equitable performance evaluation systems (12). Such supportive work environments are generally associated with reduced chronic occupational stress, lower levels of emotional exhaustion and depersonalization, and fewer manifestations of burnout (42). Conversely, nurses reporting low decent work perception often experience uncompensated overtime, inequitable performance-based pay distribution, and insufficient protection of their occupational rights. These sustained adverse working conditions progressively intensify occupational stress, ultimately diminishing work engagement and precipitating severe burnout symptoms (43, 44).

### 4.3 Mediating role of basic psychological needs

The central finding of this study lies in identifying the partial mediating role of basic psychological needs in the relationship between decent work perception and burnout, a finding that aligns closely with the basic psychological needs sub-theory of SDT. According to this framework, external environmental factors do not directly determine individuals‘ occupational behaviors and emotional states; rather, they exert their influence indirectly through the satisfaction of intrinsic psychological needs (20, 45). In our study, decent work, conceptualized as a pivotal external organizational-contextual factor, was found to correlate negatively with burnout, while exhibiting an indirect associational tendency via enhanced satisfaction of pediatric nurses' needs for autonomy, competence, and relatedness.

More specifically, a high-quality decent work environment fosters nurses' autonomy by granting them appropriate latitude to independently organize nursing procedures, thereby satisfying their need for autonomy. Hospitals that offer regular professional training and clear career advancement opportunities validate nurses' professional competence, thus addressing the need for competence (44, 46). Meanwhile, standardized humanistic management practices and harmonious relationships with both physicians and colleagues help fulfill the need for relatedness (37). When these three core psychological needs are adequately satisfied, nurses are more likely to develop stable internal work motivation, rather than being passively driven by external pressures. This internally regulated motivation, in turn, enables individuals to proactively cope with workplace challenges, withstand chronic occupational stress, and tend to report lower levels of emotional exhaustion and burnout (47). In contrast, poor decent work conditions tend to simultaneously frustrate all three basic psychological needs (40). Sustained need frustration disrupts nurses' intrinsic work motivation, precipitates negative work-related emotions, and ultimately exacerbates burnout symptoms (48, 49).

Drawing on self-determination theory, this study expands the application of basic psychological need frameworks to pediatric nurses' occupational mental health. By examining the mediating role of three psychological needs between decent work perception and burnout, this work adds empirical support for the theory within high-stress pediatric nursing contexts, offering theoretical implications for decent work research targeting specialist nursing groups. Unlike previous JDR Model dominated studies that focus on direct resource buffering effects, this paper supplements micro motivational interpretation from SDT and expands the theoretical toolbox for analyzing pediatric nurses' mental health. Still, existing literature has indicated potential reciprocal links among these study variables. Elevated burnout may correlate with poorer perceptions of decent work and less favorable psychological need experiences. Given the cross-sectional research design, we are unable to clarify the directional nature of these observed associations. Alternative explanatory mechanisms such as reverse causality therefore remain possible.

### 4.4 Practical implications for hospital nursing management

Grounded in SDT and supported by our empirical findings, this study proposes a dual-pathway approach to pediatric nursing human resource management, targeting both the external work environment and nurses' internal psychological needs. At the external level, hospitals should establish standardized occupational security systems, including evidence-based staffing, competitive compensation, and transparent performance appraisal mechanisms, all of which are empirically associated with enhanced decent work perception, a known buffer against burnout (25). At the internal level, nursing managers should balance clinical performance with psychological wellbeing by granting autonomy in decision-making and scheduling (addressing the need for autonomy), offering regular professional training and recognition programs (addressing competence), and facilitating team-building and confidential counseling services (addressing relatedness). According to SDT, satisfying these three basic psychological needs fosters intrinsic motivation and emotional resilience, which are critical for sustaining a stable and committed nursing workforce (50). The synergy between external governance and internal psychological support is likely to contribute collectively to reducing burnout prevalence among pediatric nurses.

### 4.5 Limitations and future directions

Several limitations of the present study should be acknowledged. First, the cross-sectional design restricts our ability to draw robust causal inferences regarding the relationships among the focal constructs. To rigorously test the causal ordering presupposed by our mediation model, future research would benefit from longitudinal panel designs or experimental approaches that establish temporal precedence and permit controlled manipulation of core variables. Second, the model fit indices, while not falling below conventional acceptability thresholds, were situated within a marginal range. Although this does not jeopardize the substantive interpretation of the significant path coefficients, it warrants caution in generalizing the results and suggests that these findings be viewed as preliminary pending replication in larger, better-powered confirmatory samples. Third, to mitigate the risks associated with common method bias and to strengthen causal arguments, subsequent investigations could implement multi-wave data collection or multi-source reporting, thereby introducing both temporal and procedural separation between measurements of predictors, mediators, and outcomes. Fourth, our analysis focused exclusively on the mediating role of basic psychological needs, leaving the potential influence of moderating factors, such as organizational support and psychological capital, unexamined. Future work might extend the current framework by integrating such moderators into a moderated mediation model, which could offer a more nuanced understanding of the contextual conditions shaping the psychological pathways underlying nurse burnout.

## 5 Conclusion

In conclusion, decent work perception is associated with lower levels of occupational burnout in pediatric nurses, and this association is partially accounted for by the role of basic psychological needs. Within this explanatory framework, satisfaction of basic psychological needs represents a notable pathway through which decent work perception relates to burnout. The present results imply that hospital nursing managers could implement a dual-dimensional strategy, which focuses on advancing decent workplace arrangements alongside meeting nurses' fundamental psychological demands. Such an integrated approach may effectively mitigate burnout, contribute to workforce stability, and support the sustainable, high-quality development of pediatric nursing services.

## Data Availability

The raw data supporting the conclusions of this article will be made available by the authors, without undue reservation.
